# Hand-Powered Inertial Microfluidic Syringe-Tip Centrifuge

**DOI:** 10.3390/bios12010014

**Published:** 2021-12-29

**Authors:** Nan Xiang, Zhonghua Ni

**Affiliations:** 1 School of Mechanical Engineering, Jiangsu Key Laboratory for Design and Manufacture of Micro-Nano Biomedical Instruments, Southeast University, Nanjing 211189, China; nzh2003@seu.edu.cn; 2 State Key Laboratory of Bioelectronics, Southeast University, Nanjing 210096, China

**Keywords:** inertial microfluidics, cell concentration, hand-powered, point-of-care diagnostic testing

## Abstract

Conventional sample preparation techniques require bulky and expensive instruments and are not compatible with next-generation point-of-care diagnostic testing. Here, we report a manually operated syringe-tip inertial microfluidic centrifuge (named i-centrifuge) for high-flow-rate (up to 16 mL/min) cell concentration and experimentally demonstrate its working mechanism and performance. Low-cost polymer films and double-sided tape were used through a rapid nonclean-room process of laser cutting and lamination bonding to construct the key components of the i-centrifuge, which consists of a syringe-tip flow stabilizer and a four-channel paralleled inertial microfluidic concentrator. The unstable liquid flow generated by the manual syringe was regulated and stabilized with the flow stabilizer to power inertial focusing in a four-channel paralleled concentrator. Finally, we successfully used our i-centrifuge for manually operated cell concentration. This i-centrifuge offers the advantages of low device cost, simple hand-powered operation, high-flow-rate processing, and portable device volume. Therefore, it holds potential as a low-cost, portable sample preparation tool for point-of-care diagnostic testing.

## 1. Introduction

Sample preparation is the first critical but a most time-consuming step in medical diagnostics or biochemical analysis [1,2,3]. For example, the isolation of rare cells, such as circulating tumor cells and fetal nucleated red blood cells, from complex cell populations [4,5,6] or the concentration of specific pathogens, parasites, or microorganisms from large-volume biological or environmental sample fluids [7,8,9] can significantly improve the detection sensitivity and accuracy. Currently, point-of-care testing and on-site rapid analysis pose a new challenge to sample preparation [10]. Conventional sample preparation techniques, commonly based on centrifugation [11] and fluorescence-activated cell sorting [12], rely heavily on expensive and bulky equipment, external power sources, time-consuming procedures, and experienced technicians, making them not compatible with newly emerging point-of-care diagnostic testing. In contrast, ideal sample preparation approaches for point-of-care diagnostic testing should be simple, rapid, low-cost, and portable, and thus easily applied outside the laboratory by a non-specialist without training.

As a novel approach to precisely control and manipulate cells and fluids in the microscale space, microfluidics provides new insights for on-site sample preparation owing to the advantages of miniaturization, a low device cost, small-sample consumption, and high integration [13,14]. To date, various novel microfluidic devices have been developed to realize separation [15,16], ordering [17,18], concentration [19,20], trapping [21], enrichment [22], filtration [23,24], and lysis [25,26] of cells on a single chip. In addition to the diversified application functions, continuous efforts have been made toward the development of simple, low-cost, and portable microfluidic devices for achieving effective sample preparation in resource-constrained environments at remote sites.

One option is to simplify and reduce the cost of the device fabrication process, allowing the device to be massively produced and disposed of in small clinics, homes, and field settings. In addition to well-established soft lithography [27], many new fabrication techniques have been proposed to simplify the fabrication process or to create complex non-planar structures. For example, three-dimensional (3D) printing [28,29] and laser direct writing [30,31] are new methods for directly creating innovative 3D structures in bulk materials. However, the devices fabricated by these approaches are commonly in the millifluidic size scale and require an additional step to remove the sacrificial or fused materials. Other mass production techniques include injection molding [32], roll-to-roll hot embossing [33], and xurography [34]. In addition to rapid prototyping techniques, the materials for fabricating microfluidic devices have evolved from polydimethylsiloxane [35] to low-cost paper [36], off-the-shelf tubing [37,38], and commercially available polymer films [39,40].

Alternatively, the straightforward principle of microfluidic sample preparation can be used so that the required supporting peripheral equipment can be simplified. Currently, microfluidic sample preparation can be categorized into active and passive microfluidics. Active microfluidics commonly employ external force fields, including electric [41,42], magnetic [43,44], optical [45], and acoustic [46,47] forces, to manipulate fluids or microscale objects. These methods have a high manipulation resolution but limited throughput. More importantly, complex, expensive microstructures (such as microelectrodes and interdigital transducers) and bulk field generators (such as signal generators) are still required, which prevents the miniaturization of these systems. The ideal principle for microfluidic sample preparation should be simple, low-cost, portable, and external field-free. In contrast, passive microfluidics solely applies hydrodynamic effects or specially designed microstructures to engineer fluids and cells [4,16,48] and thus are more suitable for point-of-care diagnostic testing. Among the reported passive microfluidics, inertial microfluidics has attracted increasing interest in recent years owing to its advantages of a high processing throughput, simple channel geometry, and easy operation [49,50,51,52,53]. Thus, many novel inertial microfluidic devices have been developed for various sample preparation functions, such as cell single-line ordering [54], selective trapping [55], solution exchange [56], differential separation [39,57], and efficient mixing [58].

Although passive microfluidics can work without the use of external field generators, the operation of most of these devices still relies heavily on bulk external fluid-driven systems (such as syringe pumps, peristaltic pumps, and gas-driven fluid pumping systems), which require electricity as the power source and are difficult to miniaturize. To address this limitation, various on-chip fluid pumping systems using capillary force [59], surface energy gradient [60], electroosmotic flow [61], and acoustic streaming [62] have been explored. However, the flow rates provided by these pumping systems are very low and thus are incapable of driving flows in high-flow-rate systems (such as inertial microfluidics). In turn, human power and finger actuation may be the ideal power source for driving sample fluids for point-of-care diagnostics [63,64,65], but the precise control of fluid flow generated by these low-cost power sources remains a challenge.

Great success has been achieved in simplifying device fabrication, working principle, and fluid pumping system; nonetheless, a simple, low-cost, and portable device that allows rapid sample preparation is still rarely reported. Here, we developed a hand-operated syringe-tip inertial microfluidic centrifuge (named i-centrifuge) for high-flow-rate and continuous-flow cell concentrations. The i-centrifuge consists of a syringe-tip flow stabilizer for regulating the flow generated by hand power and a four-channel paralleled inertial microfluidic device for high-flow-rate (up to 16 mL/min) cell concentrations. The integration of a flow stabilizer enables the concentration performance to be entirely independent on the operations and experiences of the user. We demonstrated the design concept, experimentally characterized the performance, and applied this novel i-centrifuge to hand-operated cell concentration. The developed i-centrifuge offers the advantages of low device cost, hand-powered simple operation, high-flow-rate processing, and portable device volume, thereby holding potential for sample preparation in resource-constrained environments.

## 2. Materials and Methods

### 2.1. Device Fabrication

For the four-channel paralleled inertial microfluidic concentrator, each channel unit was fabricated by enclosing a patterned 95 μm thick polyvinyl chloride (PVC) film with two commercially available laminating films with the assistance of a desktop laminator (LM8-330, Rayson, Foshan, China). The bonding of different channel units was achieved using double-sided tape (180 μm thick, 3M, Shanghai, China). The patterns in the PVC film, laminating films, and double-sided tapes were cut using a laser cutting system (TH-UV200A, Tianhong, Suzhou, China) equipped with a UV laser source (Awave 355-10W-30K, Advanced Optowave, New York, NY, USA).

To fabricate the elastic membrane in the syringe-tip flow stabilizer, the mixed and degassed PDMS liquid (Sylgard 184, Dow Corning, MI, USA) with a base to curing agent ratio of 10:1 was spin-coated onto a polyethylene terephthalate (PET) film and then cured at 100 °C for over 100 min. After being transferred onto the double-sided tape, the PET film was peeled off from the PDMS membrane (measured thickness of 60 μm). The holes or grooves in the PDMS membrane and double-sided tapes were cut using the abovementioned laser cutting system. The PDMS membranes with different thicknesses are also commercially available. In addition to the PDMS membrane, other commercially available elastic films could be used in the syringe-tip flow stabilizer.

The housings of the concentrator and syringe-tip flow stabilizer were directly printed in the photocurable resin (SZUV-W8001, DigitalManu, Shanghai, China) using a laser-based stereolithography 3D printer (3DSL-450S, DigitalManu, Shanghai, China).

### 2.2. Preparation of Particle/Cell Suspensions

Fluorescent particles with diameters of 10 μm (G1000B, 1% solid content, Thermal Fisher Scientific, Waltham, MA, USA) were diluted to low concentrations with phosphate-buffered saline (Sigma-Aldrich, Burlington, MA, USA). Before performing the experiments, the particle suspensions were uniformly dispersed using a vortex mixer (Thermal Fisher Scientific, Waltham, MA, USA).

Unicellular green microalgal cells (GY-H1 *Platymonas helgolandica tsingtaoensis*, Shanghai Guangyu Biological Technology, Shanghai, China) were used to characterize the concentration performance. The microalgal cells had a polydisperse size of 5–20 μm (average size: 12 μm) and a non-spherical flat shape with an average circularity of approximately 0.5. The microalgal cells were cultured in the F/2+Si medium according to the manufacturer’s instructions. After harvesting, the microalgal cells were diluted with phosphate-buffered saline to specific concentrations. In addition to the microalgal cells, human breast cancer MCF-7 cells were cultured in the high-glucose Dulbecco’s modified Eagle’s medium (DMEM, Thermo Fisher Scientific, Waltham, MA, USA) containing 10% fetal bovine serum (Thermo Fisher Scientific, Waltham, MA, USA) and 1% penicillin-streptomycin (Thermo Fisher Scientific, Waltham, MA, USA). After harvesting, MCF-7 cells were dispensed in phosphate-buffered saline (PBS, Sigma-Aldrich, Burlington, MA, USA) at specific concentrations.

### 2.3. Experimental Setup

As the 3D printed housing of our concentrator was not transparent, the device was clamped by two transparent poly(methylmethacrylate) plates to characterize the particle distribution in the four-channel paralleled concentrator. The entire device was fixed on the observation platform of an inverted fluorescence microscope (IX 71, Olympus, Tokyo, Japan). The inlet and outlets of the concentrator were connected to the syringe and centrifuge tubes using tubing. The prepared samples were loaded into a plastic syringe, which was driven by a precise syringe pump (Legato 270, KD Scientific, Holliston, MA, USA) to generate the desired flow rate. The particle distribution in the device was observed and captured using a high-speed camera (Exi Blue, Qimaging, British Columbia, Canada) under a long exposure time of 500 ms. To avoid random errors, over 100 image frames were vertically overlaid using ImageJ software (https://imagej.nih.gov/ij/ (accessed on 26 December 2021)) to create a composite image illustrating the statistical particle distribution across the channel width. When inertial focusing was achieved, a bright fluorescent stream could be clearly observed. The fluorescence intensity profile across the channel width was measured using this software. Gaussian fitting of the intensity profile was performed to obtain the full width at half maximum (FWHM) for evaluating the focusing performance.

A gas-driven flow system (Figure S1) was established to characterize the flow-stabilizing performance of the integrated syringe-tip flow stabilizer. First, a pressure controller (OB1 Base MkIII, Elveflow, Paris, France) was employed to regulate the input compressed air at a specific pressure to push the liquid out of the hermetic sample reservoir into the syringe-tip flow stabilizer. The mass of the liquid output by the syringe-tip flow stabilizer was continuously monitored using an electronic balance. Based on these data, the output volumetric flow rate of the syringe-tip flow stabilizer at a specific input pressure was calculated. Finally, the flow rate-pressure curves could be plotted.

For cell concentration application, the syringe was manually pushed, and a syringe pump was not required. During the cell concentration process, the liquids from both outlets were collected separately. The volumes and concentrations of the initial sample and the collected target samples were measured. Cell concentrations were counted using a Countess II FL automated cell counter (Thermo Fisher Scientific, Waltham, MA, USA).

## 3. Results and Discussion

### 3.1. Conceptual Design and Working Principle of i-Centrifuge

**Figure 1:**
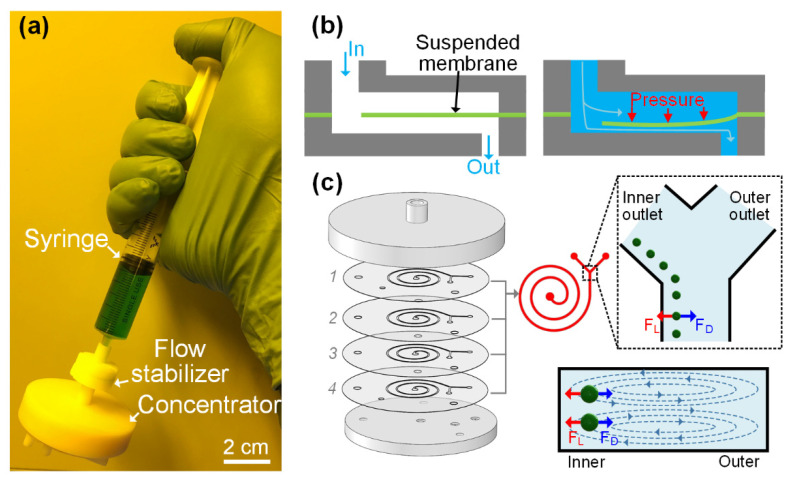
(**a**) Photograph illustrating the operation of the hand-operated syringe-tip inertial microfluidic centrifuge (i-centrifuge) for continuous-flow cell concentration. The i-centrifuge consists of a syringe-tip flow stabilizer and a syringe filter-like inertial microfluidic concentrator and can be quickly mounted onto the syringe tip via a simple press-fit connection. (**b**) Working principle of the flow stabilizer for regulating varied input liquid flow to be at a desired stable flow rate. (**c**) Structure and working principle of the syringe filter-like inertial microfluidic concentrator. Four spiral inertial microfluidic channels were integrated for achieving ultra-high throughout processing. The right part showed the cell inertial focusing principle at the position before the Y-shaped outlet system and in the cross-section of each spiral channel.

The i-centrifuge (Figure 1a), consisting of a syringe-tip flow stabilizer and a syringe filter-like inertial microfluidic concentrator, can be quickly mounted onto the syringe tip via a simple press-fit connection. The sample liquid in the syringe is first injected into the flow stabilizer under the hand-pushing operation. However, fluid flow generated by manually pushing the syringe is heavily dependent on the experience of the operator and may be highly unstable and uncontrollable. In our hand-operated system, this unstable liquid flow can be regulated to be stable at a specific flow rate using an integrated flow stabilizer. As illustrated in Figure 1b, when the liquid is injected into the flow stabilizer, the liquid will flow through the hole in the suspended membrane toward the outlet of the flow stabilizer. As the hole in the suspended membrane is small, the fluid will accumulate above the suspended membrane and apply positive pressure to the suspended membrane. The suspended membrane deforms toward the bottom wall when a positive flow pressure (∆*P*) is applied to the top of the suspended membrane, resulting in the increase in the flow resistance (∆*R*) of the formed entire flow path. The deformation degree of the elastic membrane and the resulting flow resistance of the flow path varies with the pressure applied to the membrane. Therefore, by dynamically adjusting the flow resistance of the flow path according to the input pressure, a constant output flow rate ($Q=\frac{P}{R}=\frac{P+∆P}{R+∆R}$) can be achieved under varied pressures by using the flow stabilizer. The flow regulation mechanism is passive and electricity-free, which makes this flow stabilizer especially suitable for hand-powered operations. The only requirement for actuating the flow stabilizer is to apply a pressure larger than the threshold value to induce sufficient membrane deformation for flow autoregulation. The threshold pressure is the minimum pressure when the flow-rate variation is within 5%.

After passing through the flow stabilizer, the unstable liquid flow generated by manually pushing the syringe can be automatically regulated to be at the desired value and driven into the downstream four-channel paralleled inertial microfluidic concentrator. In our concentrator, four-channel paralleled spiral inertial microfluidic channels were designed to achieve a passive cell concentration in an ultra-high-flow-rate manner. As illustrated in Figure 1c, the sample liquid flowed into each channel unit via the central inlet hole. The inlet and outlet housings (Figure S2) were used as the world-to-chip interface to quickly clamp the microfluidic concentrator. When flowing along the spiral channel at finite Reynolds numbers, the cells simultaneously suffer from the coupled effects of inertial migration and cross-sectional Dean flow [66,67], which resulted in lateral cell migration perpendicular to the main flow stream. The mechanics for inertial migration is the inertial lift force (*F*_L_) caused by the inherent inertia of microfluids. The equation of *F*_L_ can be expressed as [67]: $F_{L}=\frac{U_{m}^{2}a_{p}^{4}\rho }{D_{h}^{2}}f_{L}$, where *U*_m_ is the maximum velocity, *a*_p_ is the cell diameter, *ρ* is the fluid density, *D*_h_ is the hydraulic diameter, and *f*_L_ is the lift coefficient. The *F*_L_ is the net force of a shear-induced inertial lift force (*F*_LS_) and a wall-induced inertial lift force (*F*_LW_) [68]. The parabolic flow profile induces an *F*_LS_ to push the cell down the shear gradient toward the channel wall. The wall, in turn, induces a repulsive *F*_LW_ to push the cell away from the channel wall. In addition to *F*_L_, the cross-sectional Dean flow induces a lateral Dean drag force (*F*_D_) on cells [53]. A scaling of *F*_D_ can be expressed as [68]: $F_{D}\propto \rho U_{m}^{2}a_{p}D_{h}^{2}R^{-1}$, where the *R* is the radius of curved channels. In spiral channels, the cells are focused into a cell train at a lateral focusing position near the inner channel wall under specific flow rates. The inertial focusing position is actually the equilibration position, where the net force acting on the cells equals zero. To achieve the inertial focusing, cells need to satisfy the criterion ($a_{p}/H\geq 0.07$, where *H* is the channel height) [67]. In this work, the channel height *H* and channel width *W* were designed to be 90 μm and 500 μm, respectively. The loop number was controlled to be two. More details on the channel dimensions can be found in Table S1. According to the focusing criterion, the designed channel is able to focus the cells with diameters larger than 6.3 μm. By utilizing a Y-shaped outlet system, the focused cell train can be collected via the inner outlet, whereas the blank cell-free fluid in the outer half channel can be removed via the outer outlet. The removal of cell-free fluids can significantly reduce the volume of the target samples and thus increase the cell concentration. The passive cell concentration based on the principle of inertial focusing does not rely on external force fields and can work in a high-flow-rate, continuous-flow manner.

### 3.2. Low-Cost Four-Channel Paralleled Spiral Inertial Microfluidics for Ultra-High-Flow-Rate Processing

**Figure 2:**
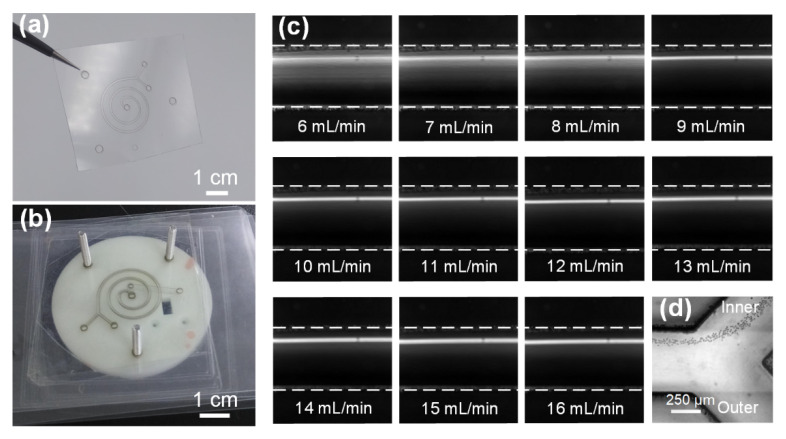
(**a**) Photograph of the single device that was fabricated in polymer films using a rapid process of laser cutting and lamination bonding. (**b**) Photograph of the four-channel paralleled device for ultra-high-flow-rate processing. Four identical channel layers were precisely aligned and vertically stacked with the assistance of locating holes in each channel layer and a corresponding fixture. (**c**) Composite images illustrating the distributions of 10 μm particles across the channel width near the outlet at the flow rates of 6–16 mL/min with an interval of 1 mL/min. White dotted lines indicate the channel walls. The upper wall is the inner wall. (**d**) Stacked bright field image illustrating the particle focusing at the Y-shaped outlet.

To lower the device cost and enable disposable use, a spiral inertial microfluidic channel designed to achieve an ultra-high-flow-rate and continuous-flow cell concentration was fabricated in low-cost polymer films using a rapid process of laser cutting and lamination bonding. Specifically, the channel geometries were patterned by cutting through grooves in a PVC film using a laser. Then, the patterned PVC film was sandwiched between two laminating films, and the through channel was enclosed using lamination (Figure 2a). This single device is highly transparent, which enables the clear observation of cell-focusing dynamics in the channels. The material cost for each device was only $0.01, which allowed its disposable use. To achieve ultra-high-flow-rate processing, four identical channel layers were precisely aligned and vertically stacked with the assistance of locating holes in each channel layer and a corresponding fixture (Figure 2b). The bonding of the different channel layers was accomplished using a patterned double-sided adhesive. The fabrication process of the four-channel paralleled device was completed within 15 min. Although four layers were stacked, the total thickness of the four-channel paralleled device was only 2 mm.

The cell concentration process using this four-channel paralleled device was based on the principle of inertial focusing. Therefore, we characterized the focusing performance of our device to better understand device physics. The 10 μm particle suspensions were pumped into the device via the inlet at the flow rates of 6–16 mL/min with an interval of 1 mL/min. Figure 2c illustrates the stacked composite images illustrating the particle distributions across the channel width before the outlet at various flow rates. It was observed that the fluorescent band gradually narrowed down and moved into the inner half channel at low flow rates of 6–8 mL/min. With a further increase in the flow rate, a clear fluorescent focusing stream was observed, indicating the formation of a focused particle train near the inner channel wall. Given the focusing of particles near the inner channel wall, the entire focused particle train could be completely removed via the inner outlet, whereas the cell-free fluids could be removed via the outer outlet (Figure 2d and Supplementary Video S1). From the abovementioned focusing phenomena, we concluded that our device could be successfully applied for cell concentration over a wide flow rate range of 9–16 mL/min. To the best of our knowledge, an operational flow rate of up to 16 mL/min is the highest value among previously reported microfluidic concentrators.

**Figure 3:**
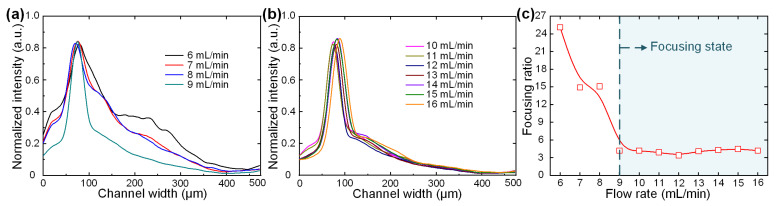
(**a**,**b**) Normalized fluorescence intensity profiles across the channel width at flow rates of (**a**) 6–9 and (**b**) 10–16 mL/min. (**c**) Focusing ratios at increasing flow rates. Particles were regarded as in focusing state in the device after the flow rate reached 9 mL/min.

To quantitatively illustrate the effect of the flow rate on particle focusing, the fluorescence intensity profiles across the channel width at various flow rates were measured (Figure 3a,b). At flow rates of 6–9 mL/min, the fluorescence intensity in the outer half channel gradually decreased with the narrowing of the fluorescent peak. At high flow rates of 10–16 mL/min, a narrow fluorescent stream was clearly observed near the inner channel wall. The lateral position of the focusing stream remained nearly unchanged during this flow rate range.

In addition to the lateral focusing position, we defined a dimensionless focusing ratio to evaluate the focusing quality (Figure 3c). The focusing ratio was defined by dividing the FWHM of the fluorescent profile by the particle diameter. It was clearly observed that the focusing ratio rapidly decreased with the increasing flow rate and then became stable after the flow rate was greater than 9 mL/min. Therefore, we concluded that the particles were in the focusing state in our device after the flow rate reached 9 mL/min. Taken together, these results demonstrate that our device is capable of focusing particles into a train with stable lateral positions and focusing qualities over a board flow rate range of 9–16 mL/min. In addition to standard-sized particles, we tested the focusing performances of microalgal cells. Supplementary Video S2 illustrates the distribution of microalgal cells before the outlet at the representative flow rate of 10 mL/min. It was found that the focusing of microalgal cells was worse than that of standard-sized particles due to the polydisperse size and irregular shape of microalgal cells. However, nearly all the microalgal cells could still be collected via the inner outlet over a board flow rate range of 9–16 mL/min. The stable performance over the board flow rate range and the sheathless and external field-free operation make this device a good choice for hand-operated applications. We next characterized the flow stabilization in different channel layers. Figure S3 illustrated the focusing performances of 10 μm particles in the top and bottom channel layers (layers 1 and 4) at the flow rate of 10 mL/min. It was found that the particles in these two layers could be focused into tight streams and completely exported by the inner outlet. The focusing positions in these two layers are slightly different due to the small flow-rate variation across different layers. We further characterized the concentration performances of devices with different layer numbers. For devices with 1–4 layers, the 100% recovery of particles could be achieved, which validates the effectiveness of our four-layer design. Further increasing the channel number would deteriorate the concentration performance (a 7.36% decrease in particle recovery was observed in devices with five layers).

### 3.3. Integration of Flow Stabilizer for Enabling Hand-Powered Operation

**Figure 4:**
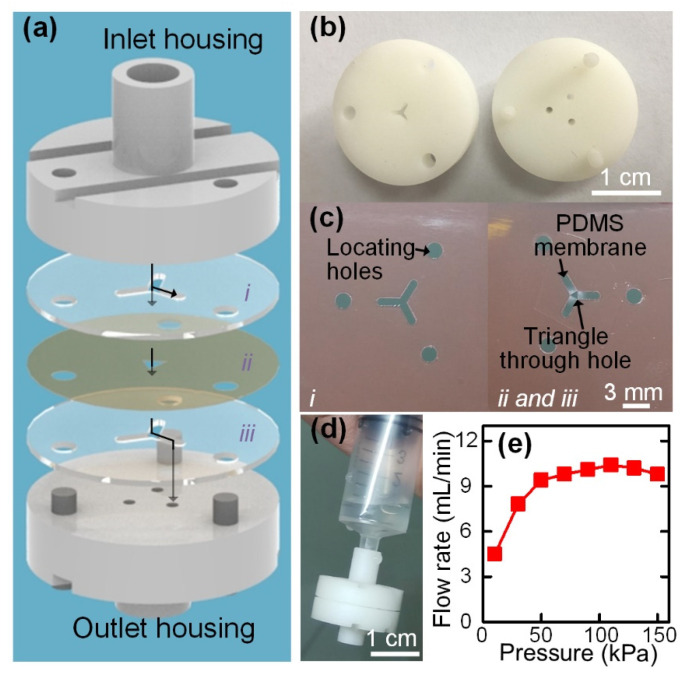
(**a**) Schematic diagram of the syringe-tip flow stabilizer containing an inlet housing, a flow stabilizing actuator (i, ii, and iii), and an outlet housing. The arrows in this figure illustrated the flow paths of one parallel flow regulator. (**b**) Photographs of the inlet and outlet housings that were fabricated by 3D printing. (**c**) Fabricated parts (i, ii, and iii) for assembling the flow-stabilizing actuator. Three parallel flow regulators were integrated in the syringe-tip flow stabilizer. (**d**) Photograph showing the prototype of the syringe-tip flow stabilizer. (**e**) Output flow rates of the syringe-tip flow stabilizer under different applied pressures.

When pushing the syringe by hand, the generated flow rates may inevitably vary during the pushing process. Although the four-channel paralleled concentrator can operate over a board flow rate range of 9–16 mL/min, it is still challenging to manually push the syringe to generate the desired flow rate within this range. Noteworthy, a flow rate that is too high or too low will deteriorate the concentration performance. To enable the concentration performance to be totally independent of the experiences and operations of the users, a syringe-tip flow stabilizer (Figure 4a–c) was integrated with the four-channel paralleled inertial microfluidic concentrator to stabilize and regulate the flow generated by hand pushing the syringe. The flow-stabilizing actuator was fabricated by stacking two layers of double-sided tapes (i and iii) and one layer of PDMS membrane (ii) in the order of tape–PDMS–tape (Figure 4a). The locating holes in each layer were used to precisely align the different layers. The two layers of double-sided tape were patterned with three branching channels. After transferring the PDMS membrane onto one surface of the double-sided tape, a triangular through hole was cut on the PDMS membrane in the central region of the three branching channels (Figure 4c and Figure S4).

By stacking the tape–PDMS–tape, a portion of the PDMS membrane was suspended over the three branching channels, forming three parallel flow regulators. In the current design, three flow regulators were radially arrayed to increase the output stable flow rate, and these flow regulators shared the same inlet. The working principle of each parallel flow regulator is described in Figure 1b. When the sample is injected from the inlet, apart from flowing through the central hole, part of the fluids will be distributed into the upper branching channels with dead ends. The fluids in the upper branching channels above the membrane apply positive pressure on the suspended PDMS membrane, resulting in the deformation of the PDMS membrane toward the cavities of the lower three branching channels. As the fluids will flow along the lower branching channels to the outlets, the deformation of the PDMS membrane into the lower branching channels significantly increases the resistance of the flow paths. By dynamically regulating the flow resistance of the flow path according to the input flow pressure, a constant output flow rate can be achieved under a hand-powered operation. The only requirement for actuating the flow stabilizer is to apply a pressure larger than the threshold value. The integration of three parallel flow regulators enables the syringe-tip flow stabilizer to provide an ultra-high driving flow rate with small deviations.

Next, we fabricated a prototype of the syringe-tip flow stabilizer (Figure 4d) that could output a flow rate within the optimal working flow rate range of the four-channel paralleled inertial microfluidic concentrator. The detailed design parameters for the syringe-tip flow stabilizer are listed in Table S2. The detailed mechanisms behind these design parameters can be found in our previous study [69]. In the current work, we combined three parallel flow regulators (each with a constant flow rate of 3.50 mL/min) in a syringe-tip flow stabilizer for outputting an ultra-high driving flow rate and then integrated the syringe-tip flow stabilizer with the syringe filter-like inertial microfluidic concentrator for concentrating the cells from large-volume samples.

After fabrication, we set up a gas-driven flow system (Figure S1) to characterize the flow-stabilizing performance of the syringe-tip flow stabilizer. The analysis of the output flow rates of the flow stabilizer with increasing applied pressure showed that the pressure first increased with increasing pressure and then became constant at 9.95 mL/min after the pressure increased to more than 50 kPa (Figure 4e). A small deviation in the flow rate after achieving a stable output was only 3.6%. Under a flow rate of about 10 mL/min, the injection of 10 mL of the sample could be completed within 1 min, which is affordable for hand-pushing operations. The low threshold pressure for outputting a constant flow rate could be easily provided by hand pushing the syringe (pressure of 63–150 kPa measured by different adult operators, *N* > 5). Therefore, the operators only need to continuously push the syringe at their own comfortable speed.

### 3.4. Hand-Operated Cell Concentration

Finally, we integrated the syringe-tip flow stabilizer with our four-channel paralleled inertial microfluidic concentrator for hand-operated cell concentrations. To set up the integrated device, a syringe was first plugged into the inlet of the flow stabilizer, while the outlet of the flow stabilizer was directly connected to the central inlet of the concentrator. The press-fit connection enables the quick assembly or disassembly of these components without leakage, and the integrated device can be easily operated using a single hand. The suspensions of microalgal cells, 10 μm standard-sized particles, and MCF-7 tumor cells with initial concentrations of about 4 × 10^5^ counts/mL were prepared and employed in this experiment. Five volunteers, none of whom underwent any operation training before the test, were invited to operate the syringe to inject the samples into the integrated device. The unstable flow rate generated by manually operating the syringe could be regulated to be the desired stable value after passing through the syringe-tip flow stabilizer. Then, the stable sample flow could power inertial focusing of cells in the concentrator, achieving the cell concentration at the optimal performance. To quantitatively characterize the performance of the integrated device for cell concentration under hand-powered operation, the liquid volumes and cell concentrations of the initial samples and those collected from both outlets were analyzed. We defined two dimensionless parameters: recovery efficiency (RE) and concentration factor (CF). RE was calculated by dividing the cell number ($n_{\mathrm{target}}$) in the target sample collected from the inner outlet by the total cell number in all outlets ($n_{\mathrm{total}}$), and CF was calculated by dividing the cell concentration ($c_{\mathrm{target}}$) of the target sample collected from the inner outlet by the cell concentration ($c_{\mathrm{initial}}$) of the initial sample.

**Figure 5:**
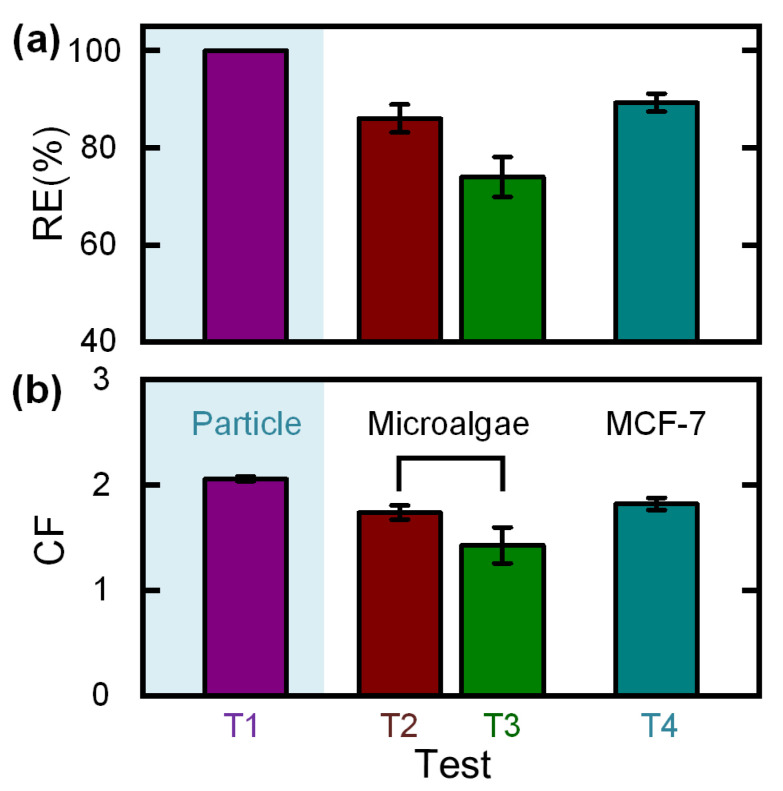
(**a**) Recovery efficiency (RE) and (**b**) concentration factor (CF) of 10 μm standard-sized particles, microalgal cells, and MCF-7 tumor cells under different experimental tests (T1−T4). Tests T1, T2, and T4 were performed with the integrated device using 10 μm standard-sized particles, microalgal cells, and MCF-7 cells, respectively. Test T3 was a control experiment on the concentration of microalgal cells using a device without a flow stabilizer. All the experiments were repeated five times under the hand-operated mode. The error bar in this figure denoted the standard deviation.

Under the hand-operated mode, an RE of 100% and a CF of 2.06 ± 0.02 was achieved for 10 μm particles (T1 in Figure 5). For microalgal cells, an RE of 86.1 ± 2.9% and a CF of 1.74 ± 0.07 was obtained (T2 in Figure 5). Moreover, for MCF-7 tumor cells, an RE of 89.3 ± 1.8% and a CF of 1.82 ± 0.06 was obtained (T4 in Figure 5). The concentration performances of microalgal cells and MCF-7 tumor cells were found to be worse than of standard-sized particles because of the highly polydisperse cell sizes.

For comparison, we performed a control experiment on the concentration of microalgal cells using a device without a flow stabilizer (T3 in Figure 5). It was noticeable that the RE decreased to 74.1 ± 4.1%, and the CF decreased to 1.43 ± 0.17 due to the unstable flow rate generated by varied pushing operations (such as pushing at non-uniform speeds). As the syringe-tip flow stabilizer can provide a constant flow rate, the use of our flow stabilizer can make the concentration performance of the downstream concentrator totally independent of the user-pushing operations. The low threshold pressure (50 kPa for the current device) required to drive the flow stabilizer to output a constant flow rate can be easily provided by any adult operator.

To deal with samples with low initial cell concentrations at the level of 10^4^ counts/mL, a multistep serial concentration was performed by reinjecting the collected target sample into the integrated device. With the 10 mL sample being reduced to less than 1 mL, the concentrations of microalgal cells in the target samples gradually increased from 0.586 × 10^5^ to 6.96 × 10^5^ counts/mL. After the multistep serial concentration, a total CF of 11.9 was achieved. The entire concentration process (including the sample reloading time) was completed within 5 min. Through observation of the migration of microalgal cells under a microscope, it was found that the cells remained alive and active after running through the integrated device. For MCF-7 cells, the cell viability was evaluated by Trypan blue exclusion. As illustrated in Figure S5, the cells remained alive after being processed with the integrated device. We next explored the effect of initial cell concentration on concentration performance using the microalgal cells. When the initial cell concentrations were below 2.4 × 10^5^ counts/mL, an RE approaching 95% and a CF of about 2 could be achieved. Further increasing the initial cell concentration to be 5×10^5^ counts/mL, the RE and CF decreased to be 70.8% and 1.35, respectively. The deterioration of concentration performance was caused by the heavy cell interactions at an increased cell concentration. In addition to microalgal cells and MCF-7 cells, the i-centrifuge can be applied to concentrate various other cells (e.g., the pre-sorted rare circulating tumor cells in clinical samples and the pathogenic bacterium in environmental samples). Through adjusting the outlet system, our i-centrifuge is possible to separate different-sized particles/cells according to their differential focusing positions. Centrifugation is the gold standard for cell concentration but requires electricity and an expensive centrifuge. As compared with the centrifuge, our i-centrifuge offers the advantages of electricity-free hand-operated operation, a low device cost, and a small footprint, which makes our device especially suitable for cell concentration in the field or other resource-poor settings.

## 4. Conclusions

In this study, we describe a novel integrated syringe-tip inertial microfluidic centrifuge (i-centrifuge) consisting of a syringe-tip flow stabilizer and a four-channel paralleled inertial microfluidic concentrator. The key components of the i-centrifuge were fabricated with low-cost polymer films and double-sided tape using a rapid nonclean-room process of laser cutting and lamination bonding, which enables their disposable use. Moreover, the i-centrifuge can be directly mounted onto the syringe tip via a quick press-fit connection and can be operated by hand power independent of the experiences and operations of the user. Noteworthy, the unstable and undesired liquid flow generated by the hand-pushing syringe can be regulated to be at a specific flow rate with the assistance of an integrated syringe-tip flow stabilizer. Then, the regulated liquid flow was used to drive the four-channel paralleled inertial microfluidic concentrator to enable high-flow-rate (up to 16 mL/min) cell concentration. Overall, we experimentally demonstrated the working mechanism and performance characterization of the i-centrifuge, successfully applying it to the hand-operated concentration of particles and cells. In summary, the i-centrifuge offers the advantages of a low device cost, simple hand-powered operation, high-flow-rate processing, and portable device volume. Therefore, it holds potential as a low-cost, portable sample preparation tool for point-of-care diagnostic testing.

## Data Availability

The data presented in this study are available on request from the corresponding author.

## Supplementary Materials

The following are available online at https://www.mdpi.com/article/10.3390/bios12010014/s1, Figure S1: The gas-driven flow system for characterizing the performance of our syringe-tip flow stabilizer. The compressed air was regulated via a computer-controlled pressure controller to generate a specific pressure for driving the liquid in the hermetic sample reservoir. The values of pressures were monitored and recorded using the software. The liquid then flowed through our syringe-tip flow stabilizer, and the mass of the output fluid was continuously monitored using an electronic balance. On the basis of these data, the stable output flow rates at specific pressures could be calculated. Figure S2: Images illustrating the designs and structures of inlet and outlet housings. Figure S3: Focusing performances of 10 μm particles in the top and bottom channel layers. Figure S4: Assembly and fabrication process of the PDMS membrane. The PDMS membrane was transferred onto one side of the patterned double-sided tape using a polyethylene terephthalate (PET) film. After being transferred onto the double-sided tape, the PET film was carefully peeled off, and a triangle through hole was cut on the PDMS membrane at the central region of the three branching channels. Figure S5: Image illustrating the cell viability. Table S1: Dimensions of spiral channels. Table S2: Dimensions of the three parallel flow regulators in the syringe-tip flow stabilizer. Video S1: A video illustrating the particle focusing at the Y-shaped outlet. Video S2: A video illustrating the distribution of microalgal cells at the Y-shaped outlet.
