# Supplementary material for: Hand-Powered Inertial Microfluidic Syringe-Tip Centrifuge
Source: Biosensors (Basel). 2021 Dec 29;12(1):14. doi: 10.3390/bios12010014 (PMC8774109; doi:10.3390/bios12010014)
Supplement: Supplementary file 1 [file biosensors-12-00014-s001.zip › biosensors-1505966-sup.pdf]

biosensors

Article

Hand-Powered Inertial Microfluidic Syringe-Tip Centrifuge

Nan Xiang [1,2,*] and Zhonghua Ni [1,2]

1 School of Mechanical Engineering, Jiangsu Key Laboratory for Design and Manufacture of Micro-Nano Biomedical Instruments, Southeast University, Nanjing, 211189, China; nan.xiang@seu.edu.cn (N.X.); nzh2003@seu.edu.cn (Z.N.)
2 State Key Laboratory of Bioelectronics, Southeast University, Nanjing, 210096, China
* Correspondence: nan.xiang@seu.edu.cn

Citation: Xiang, N.; Ni, Z. Hand-Powered Inertial Microfluidic Syringe-Tip Centrifuge. *Biosensors* **2021**, *11*, 14. https://doi.org/10.3390/bios12010014

Received: 27 November 2021
Accepted: 28 December 2021
Published: date

Publisher's Note: MDPI stays neutral with regard to jurisdictional claims in published maps and institutional affiliations.

Copyright: © 2021 by the authors. Submitted for possible open access publication under the terms and conditions of the Creative Commons Attribution (CC BY) license (https://creativecommons.org/licenses/by/4.0/).

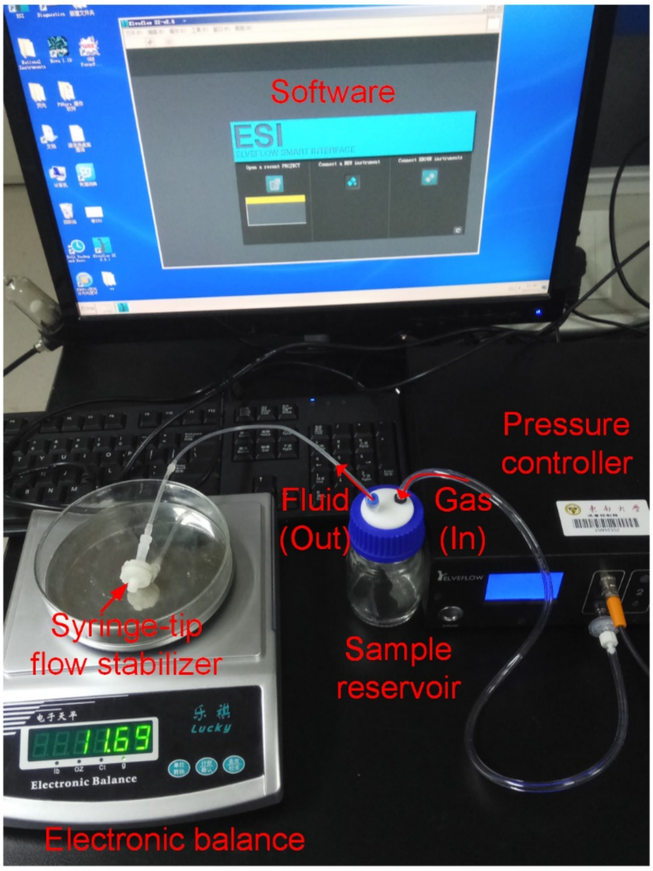

Figure S1. The gas-driven flow system for characterizing the performance of our syringe-tip flow stabilizer. The compressed air was regulated via a computer-controlled pressure controller to generate a specific pressure for driving the liquid in the hermetic sample reservoir. The values of pressures were monitored and recorded using the software. The liquid was then flow through our syringe-tip flow stabilizer and the mass of the output fluid was continuously monitored using an electronic balance. On the basis of these data, the stable output flow rates at specific pressures could be calculated.

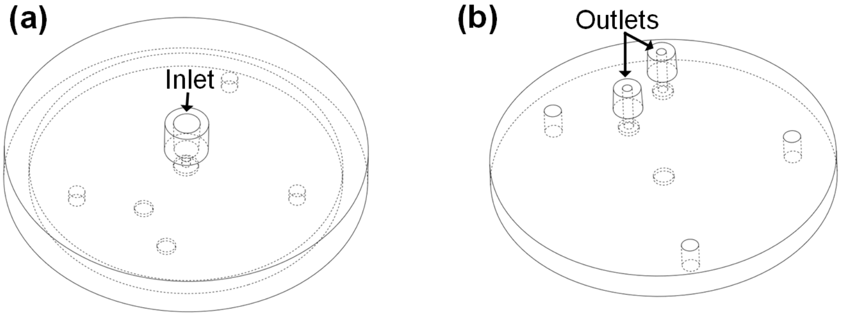

Figure S2. Images illustrating the designs and structures of inlet (a) and outlet (b) housings.

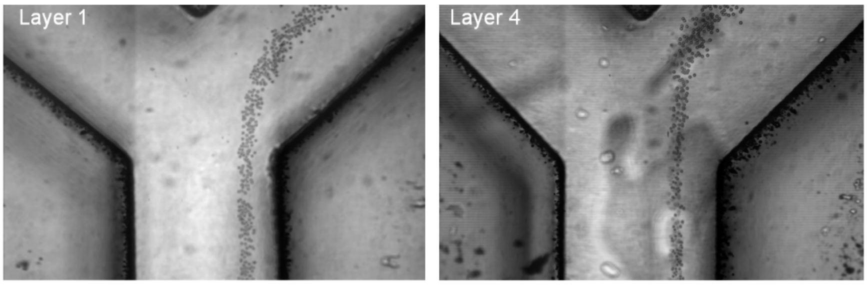

Figure S3. Focusing performances of 10 μm particles in the top and bottom channel layers (layers 1 and 4) at the flow rate of 10 mL/min.

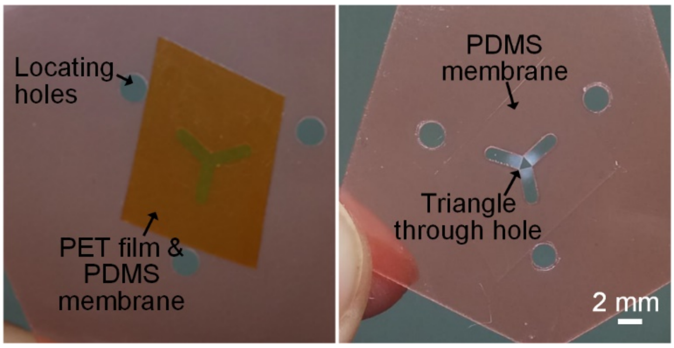

Figure S4. Assembly and fabrication process of the PDMS membrane. The PDMS membrane was transferred onto one side of the patterned double-sided tape using a polyethylene terephthalate (PET) film. After being transferred onto the double-sided tape, the PET film was carefully peeled off and a triangle through hole was cut on the PDMS membrane at the central region of the three-branching channels.

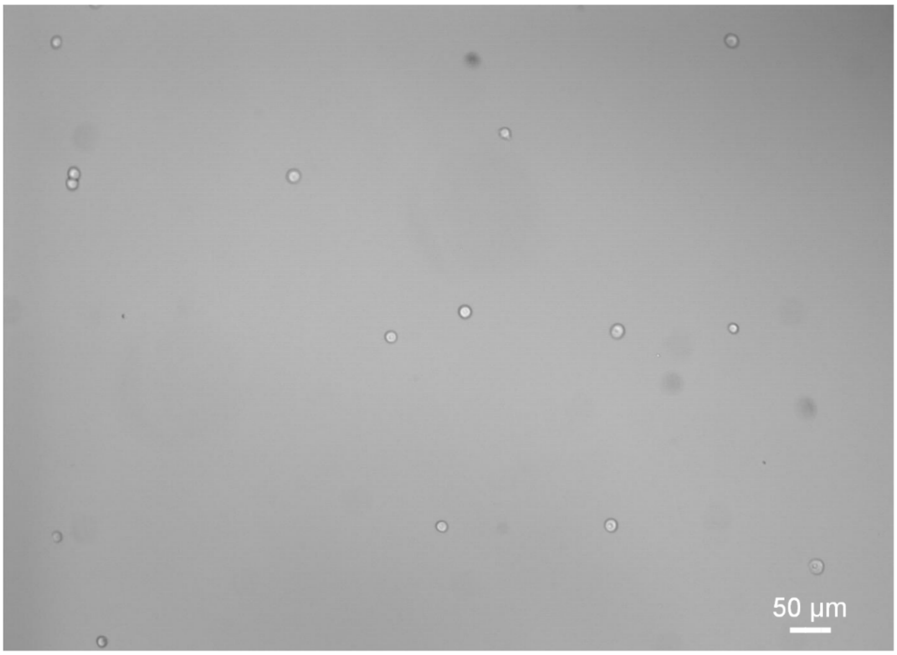

Figure S5. Image illustrating the viability of cells after being processed with the integrated device by using the Trypan blue exclusion test.

Table S1. Dimensions of spiral channels.

Dimensions	Values
Channel height (*H*)	90 µm
Channel width (*W*)	500 µm
Initial radius of spiral channel	2 mm
Loop number	2 loops
Distance between the adjacent loops	3.25 mm

Table S2. Dimensions of the three parallel flow regulators in the syringe-tip flow stabilizer.

Dimensions	Values
Length of each branching channel	3.19 mm
Width of each branching channel	1 mm
Angle between two adjacent branching channels	120°
Height of each branching channel<br>(i.e., thickness of double-sided tapes)	180 µm
Thickness of PDMS membrane	60 µm
Side length of triangle through hole	1 mm
